# Comparative study of cardio-protective effect of metformin versus dapagliflozin in experimentally induced myocardial infarction in diabetic rats

**DOI:** 10.1186/s40360-026-01110-6

**Published:** 2026-03-21

**Authors:** Dina Elhantery, Somaia Abdullatif Mokbel, Abdelaziz M. Hussein, E. M. Tayee, Sara El Desouky, Ahmed Mohammed Taha

**Affiliations:** 1 Department of Medical Pharmacology, Faculty of Medicine, Helwan University, Cairo, Egypt; 2 Department of Clinical Pharmacology, Faculty of Medicine, Mansoura University, Mansoura, Egypt; 3 Department of Medical Physiology, Faculty of Medicine, Mansoura University, Mansoura, Egypt; 4 Medical Experimental Research Center, Faculty of Medicine, Mansoura University, Mansoura, Egypt

**Keywords:** Metformin, Dapagliflozin, Type II diabetes mellitus, Myocardial infarction-isoprenaline

## Abstract

**Objective:**

To study the potential cardioprotective role of dapagliflozin in comparison to metformin in experimentally- induced myocardial infarction in rats with type 2 diabetes mellitus (T2DM).

**Materials and Methods:**

This study was comparative study, which was housed at the animal house, Mansoura Experimental Research Center (MERC). Fifty adult male albino rats were included in this study and there were further randomly divided into Group-I: (Normal), Group –II: rats received Streptozotocin (STZ) for induction of type 2 DM. This group was subdivided into (4) equal subgroups as the following: Group –IIa: (DM), Group-IIb: (DM+ISO), Group–IIc: (DM+ISO+Metformin), Group-IId: (DM+ISO+DAPA). The outcomes of the study were measured by ECG recording, serological study which included Interleukin (IL)-10, IL6, and tumor necrosis factor (TNF)-α, troponin, lipid profile and blood glucose and histopathological examination of isolated hearts.

**Results:**

Dapagliflozin show better cardioprotective effect than Metformin with lower IL-10, IL-6, TNF, CTNI and Troponin C levels than Metformin with statistically significant difference (*p* < 0.001*). The percentage of area of edema and degeneration is the lowest in controls followed by DM+ISO+DAPA group then DM+ISO+Metformin group when compared with DM and DM+ISO groups which revealed the cardio protective effect of DAPA and Metformin with inter-groups statistically significant difference (*p* < 0.001*)

**Conclusion:**

Dapagliflozin has cardioprotective effects against myocardial infarction in type 2 diabetic rats. This effect might be due to its antifibrotic and antiarrhythmic effects. However, Metformin did not show any antifibrotic or anti-arrhythmic effects.

## Introduction

Among the fastest-growing diseases globally, diabetes is expected to impact 693 million adults by 2045 [1]. Stroke, Lower limb amputation and Acute myocardial infarction (MI) are among the prevalent severe complications associated with diabetes that contribute significantly to the disease’s expense and personal burden. The burden of diabetes-related complications has increased significantly in recent decades, corresponding with the notable rise in the prevalence of diabetes worldwide [2].

The major cardiovascular diseases associated with diabetes like peripheral artery disease, coronary artery disease, heart failure, stroke, and ischemic heart disease can lead to death of about 50% of patients with type II diabetes mellitus. Type 2 diabetes is characterized by abnormal lipid metabolism, hyperglycemia, and insulin resistance [2, 3]. Crucially, a larger relative risk of cardiovascular events is linked to insulin resistance. Elevated blood glucose is strongly associated with the risk of macrovascular and microvascular issues in individuals with type 2 diabetes [3].

Some modern anti-diabetic medications have an impact on significant adverse cardiovascular events, according to recent results from cardiovascular outcome trials. Among these medications include sodium-glucose co-transporter (SGLT2) inhibitors and agonists of the glucagon-like peptide-1 (GLP-1) receptor [4].

Metformin, which is frequently used for type-2 diabetes, stimulates hepatic and muscle adenosine monophosphate activated protein kinase (AMPK), which helps insulin to be more sensitive. By inhibiting nuclear factor kappa-light-chain-enhancer of activated B cells (NFκB), metformin may lessen inflammation via both independent and AMPK-dependent mechanisms. It was demonstrated to suppress the inflammatory response in rat smooth muscle cells through the tensin homolog pathway and AMPK-phosphatase. Moreover, Nitric oxide (NO), prostaglandin E2, and pro-inflammatory cytokines such interleukins (IL), IL-1β, IL-6, and tumor necrotic factor-α are all decreased by metformin because it prevents NFκB activation in macrophages [5].

A relatively recent family of anti-hyperglycemic medications for the treatment of type 2 diabetes are sodium-glucose cotransporter-2 (SGLT2) inhibitors. By blocking the proximal convoluted tubule’s high-capacity glucose transporter SGLT2, these substances lower blood glucose levels without the help of insulin by reducing the kidneys’ reabsorption of glucose and facilitating its excretion in the urine [6].

In contrast to metformin, this study aimed to determine dapagliflozin’s possible cardioprotective benefits in experimentally- induced myocardial infarction in rats with type –II diabetes mellitus.

## Materials and Methods

### Drugs

1. Metformin: It was provided in the form of tablets **(Glucophage®),** obtained from **(Mina pharm company).** The tablets were crushed and dissolved in saline. It was given intragastric by gavage in a dose of 100 mg/kg/day.
2. Dapagliflozin: It was provided in the form of tablets **(Forxiga®)** obtained from **AstraZeneca pharmaceuticals LP**. The tablets were crushed and dissolved in saline. It was given intragastric by gavage in a dose of 1 mg/kg/day.
3. Streptozotocin **(zanosar):** It was obtained from **(Keocyt).** It was given intra-peritoneal injection by single dose of 45 mg/kg
4. Isoprenaline **(isolin):** It was obtained from **(SAMARTH).** It was given to rats in a dose of 150 mg/kg subcutaneous injection in anterior abdominal wall.

### Materials

- **Animals used in experiments and groups:**

This study comprised 50 adult male albino rats who were 12 weeks old and weighed between 200 and 250 grams on average. Following obtaining local approval, they were kept in the animal house at M**ansoura Experimental Research Center (MERC)**, This had a 12-hour light/dark cycle and a regulated temperature of 22 ± 2 °C. Every subgroup received grains and unlimited food while being housed at room temperature in a different wire cage. The standard cage size was sufficient to allow for each rat’s typical social behavior. With ventilation and ambient humidity, no more than six rats per cage were the ideal number of animals. Every technique was carried out in compliance with ethical standards that were authorized by **Mansoura University’s Animal Ethics Committee and Faculty of Medicine’s Helwan University Research Ethics Committee (88–2023).**

- **Induction of Diabetes millets (DM):**

Single dose intra-peritoneal injection of streptozotocin (50 mg/kg). Serum glucose concentration was measured after 72 hours and on the 7th day of start of experiment. Animals were deemed diabetic and included in the experiment if their non fasting glucose levels were greater than 250 mg/dl, others were excluded [7].

- **Induction of myocardial infarction:**

On days 14 and 15, a subcutaneous injection of isoprenaline at a dose of 80 mg/kg was and Eosin Stain for routine histologicaadministered to the anterior abdomen wall [5].

• **Experimental Design:**

A. **The following groups of rats were randomly selected:**
B. **Group- I (Normal Control) (10 rats):** received 0.5 ml saline by oral gavage for 15 consecutive days.
C. **Group –II (40 rats):** rats received Streptozotocin (STZ) for induction of type 2 DM. This group was subdivided into (4) equal subgroups as the following:

- **Group –IIa:** consisting of 10 diabetic rats that were given 0.5 ml of saline orally by gavage for 15 days in a row, acting as a positive diabetic control.
- **Group-IIb:** 10 diabetic rats were used as a diabetic-myocardial infarction control group. They were given 0.5 ml of saline orally by gavage for 15 days in a row, and on days 14 and 15, they got a subcutaneous injection of isoprenaline (ISO) at a dose of 80 mg/kg in the anterior abdominal wall.
- **Group –IIc:** comprised 10 diabetic rats, were received metformin in a dose of 150 mg/kg/day for 15 consecutive days in addition, they were received isoprenaline in a dose of (80 mg/kg) subcutaneous injection in anterior abdominal wall on days 14^th^ & 15^th^.
- **Group- IId:** included ten diabetic rats that were given a subcutaneous injection of isoprenaline at a dose of 80 mg/kg in the anterior abdominal wall on days 14 and 15 and dapagliflozin (DAPA) at a dose of 1 mg/kg each day for 15 days in a row.

All the animals were sacrificed by thiopental sodium overdose.

- **Measurements:**

ECG (electrocardiogram) recording:

After the experimental period was over, the electrocardiogram (ECG) was analyzed in each group at the Medical Physiology department of the Mansoura Faculty of Medicine, Mansoura University. Ketamine (80 mg/kg i.p.) and xylazine (16 mg/kg i.p.) were used to anesthetize the rats. Disposable vinyl electrodes, three electrodes per rat, the Biopac electrode lead set ×2, the Biopac student lab system (software BSL 3.7.5), and the data collection device MP36 were used to capture the ECG.

Serological study

Blood samples were collected after scarification of animals by venous puncture for assessment of:

1. IL-10 using Rat Interleukin 10 (IL-10) ELISA Kit.
2. IL6 using The Quantikine® Rat IL-6 ImmunoassayKit.
3. TNF-α using Rat TNF-α ELISA Kit (CUSABIO).
4. Troponin using Rat Cardiac Troponin I SimpleStep ELISA® Kit.
5. Triglycerides using Triglycerides GPO-POD. Liquid (SPINREACT).
6. LDL using LDL Cholesterol D Enzymatic colorimetric. Liquid (SPINREACT).
7. HDL using HDL Cholesterol P Reactive precipitate (SPINREACT).
8. blood glucose using Glucose GOD-POD. Liquid (SPINREACT).

All biochemical tests were conducted in the **animal house, Mansoura Experimental Research center (MERC)**

Heart isolation and histopathological examination

**Fig. 1:**
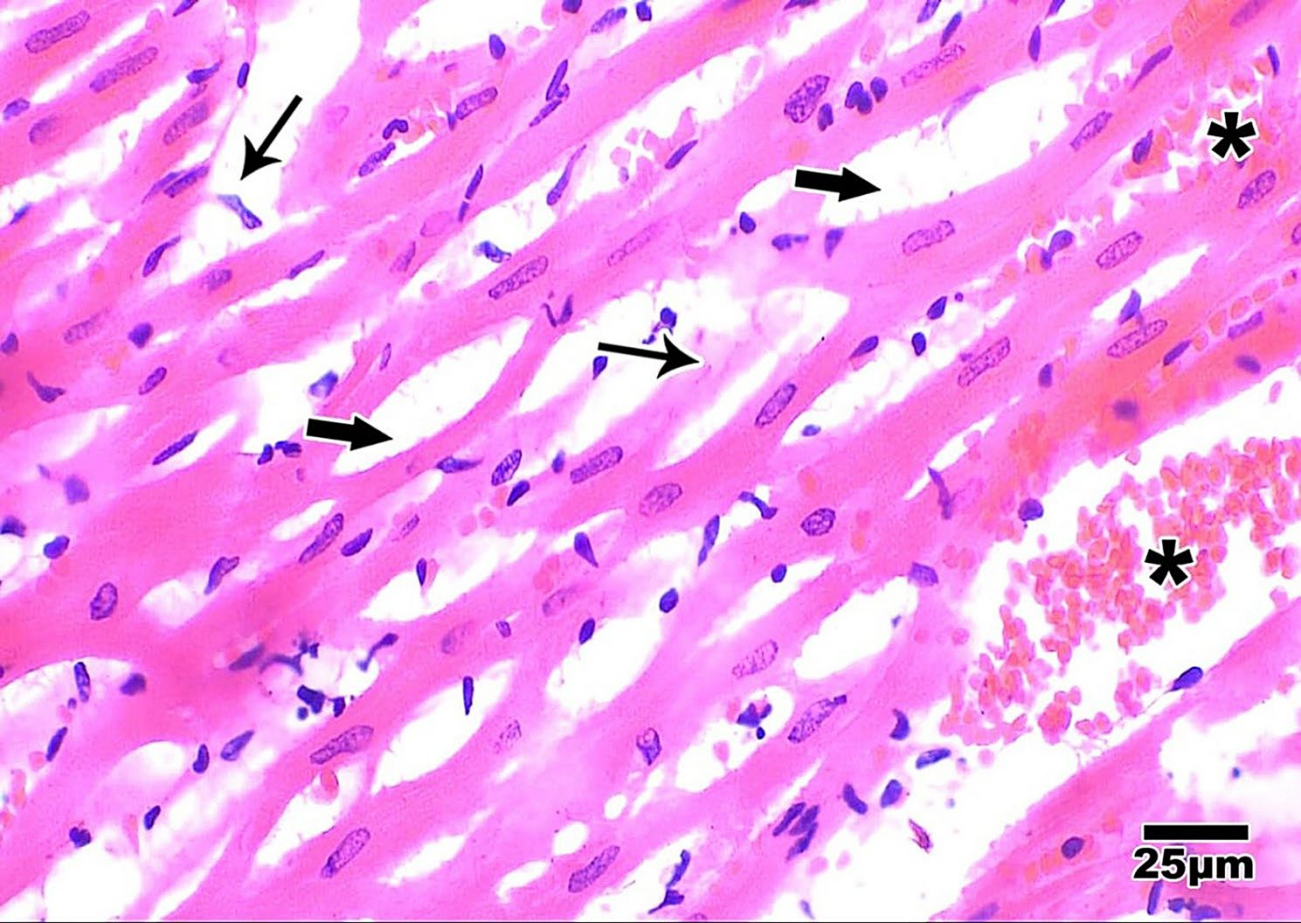
Original Hx and E-stained image

**Fig. 2:**
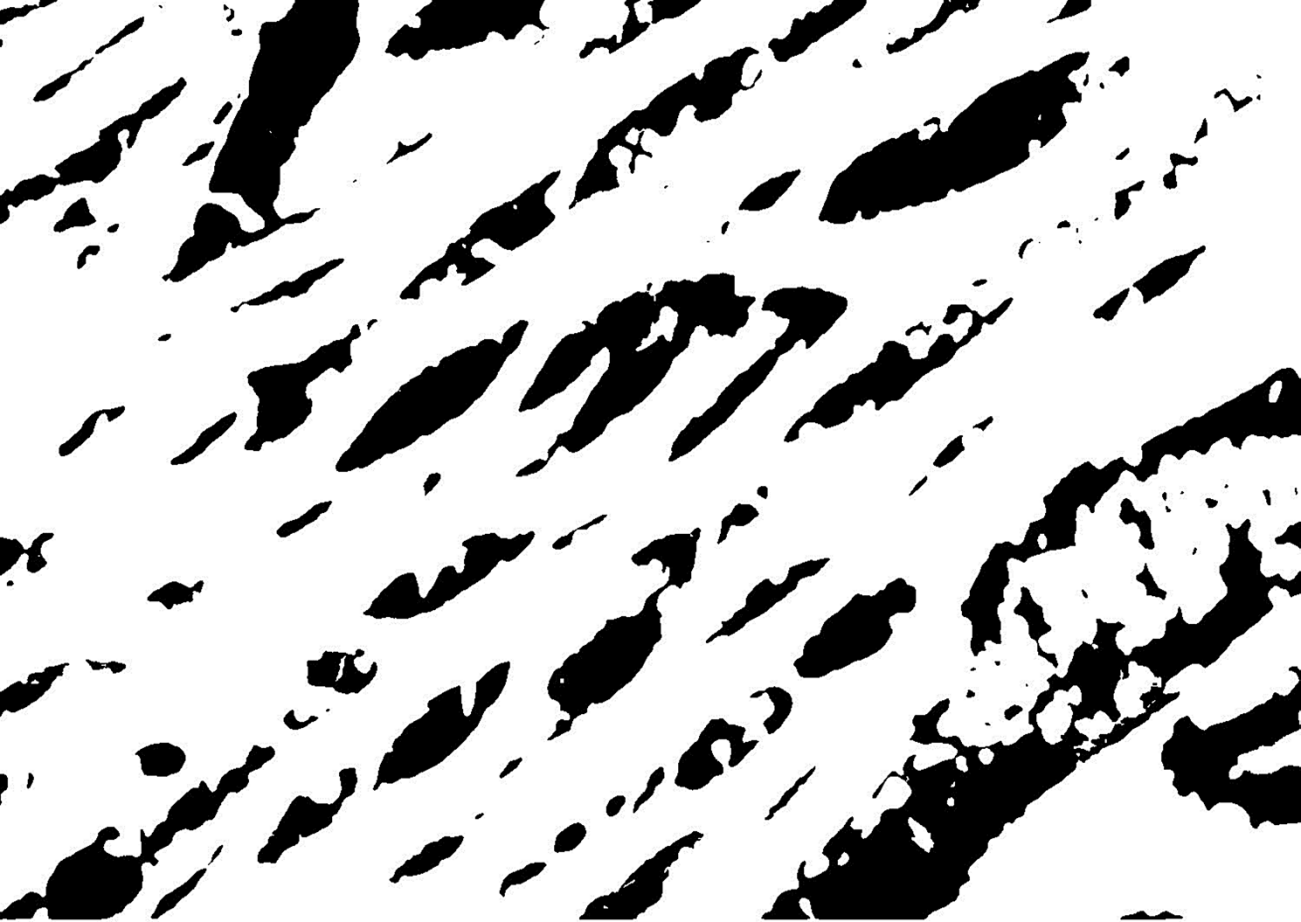
Shaded white component of the image with elimination of all other colors using photoshop CS6

A) Heart isolation: The thoracic cavity was cut with a surgical scalpel, and the heart was separated from the major blood veins and mediastinum.
B) Staining: Following cardiovascular risk that is mostly unrelated to their benefits on decreasing bloodgross examination, Small pieces (0.5x0.5x0.5 cm), were fixed in 10% neutral-buffered formalin and paraffin sections of 3–5 µm thickness were prepared and stained with Hematoxylin and Eosin Stain for routine histological examination [8].
B) **Calculation of Area of Degeneration:** by Computer Assisted digital image analysis (Digital morphometric study) Slides were photographed using Olympus® digital camera installed on Olympus® microscope with 1/2 X photo adaptor, using 40X objective. The result images were analyzed on Intel® Core I7® based computer using Video Test Morphology® software (Russia) with a specific built-in routine for area, % area, measurement, object counting and contact Angle.
C) **Methods for calculating the area percentage of edema** (Extra-cellular matrix) (×400) in the cardiac muscle of Hx and E-stained sections. Five different non-overlapping randomly selected fields at a magnification of 400 were examined in 5 slides from each group. The total area was measured after selection by adjusting the color threshold while excluding the background; thereafter, the white stained area was selected and measured to calculate the area percentage as white-stained area/total area × 100 Figs. 1 and 2.

### Method for calculating the area percentage

Using a 40X lens and a 1/2 X photo adapter, an Olympus® digital camera was mounted on an Olympus® microscope to take pictures of the slides. Video Test Morphology® software (Russia) with a built-in procedure for area, % area, measurement, object counting, and contact Angle was used to analyze the resultant images on an Intel® Core I7®-based computer.

Methods for calculating the area percentage of edema (Extra cellular matrix) (×400) in the cardiac muscle of Hematoxylin (Hx) and Eosin (E)-stained sections

Five slides from each group were analyzed at a 400x magnification using five distinct, non-overlapping fields that were randomly chosen. The white-stained region was chosen and measured to determine the area percentage, which is equal to white-stained area/total area × 100. The whole area was then measured by altering the color threshold while omitting the background.

**Step 1:** The original image was transformed into a black and white scale image using the Photoshop ® CS6 application.

**Step 2:** All the image’s color components were removed using the Photoshop ® CS6 application, leaving only the black variations of the white color component.

**Step 3:** Using the Image J application, the image was processed, transformed into a threshold image by following the instructions in the program’s menu (Image > modify > threshold), and the threshold was changed as needed.

**Step 4:** The Image J program’s menu item (analyze > analyzed particles) was used to determine the area % of edema.

**Step 5:** A text file with the results was exported.

### Technique for calculating the quantity of degenerated cells in sections labeled with Hx and E

For each group, five distinct non-overlapping fields were randomly chosen and seen at a magnification of 400; only cells that had degenerated were counted. The Image J application was used to count the cells. The program’s menu item (plugins > analyze > cell counter) was used to do this. Clicking on each cell allowed for the counting of all the desired cells.

#### Statistical study

To analyze the data, Version 26 (SPSS Inc., PASW statistics for Windows) of the SPSS software was used. Numbers and percentages were used to describe the qualitative data. To characterize quantitative data, mean± was utilized. standard deviation for normally distributed data measured by the Shapiro-Wilk test. The results’ significance was assessed at the 0.05 level. More than two independent groups were compared using the One-Way ANOVA test, and pairwise comparisons were detected using the Post Hoc Tukey test.

## Results

### Results of ECG changes between different groups

**Fig. 3:**
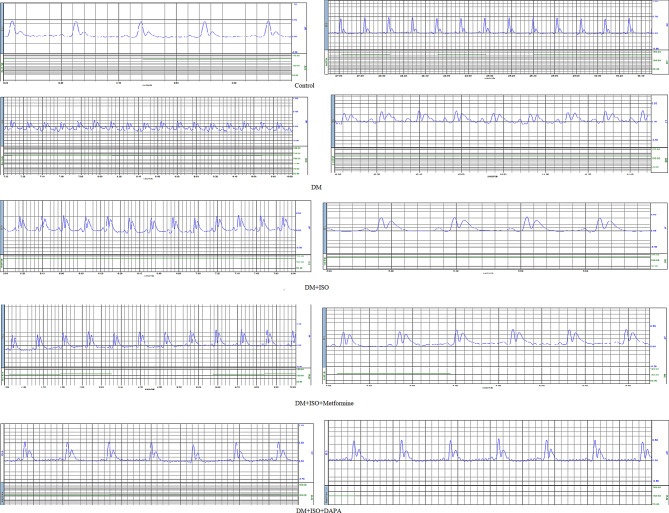
EGC finding among study groups

The ECG changes between the 5 groups include change on heart rate, PR interval time, QT interval time R wave amplitude and T wave amplitude were listed in Table 1 and Fig. 3.

**Table 1:** ECG (electrocardiogram) recording of studied groups

	Normal N = 10	DM N = 10	DM+ISO N = 10	DM+ ISO + Metformin N = 10	DM+ISO+DAPA N = 10	Test of significance
**Heart rate (beat/min)**	226.07 ± 20.41	321.7 ± 23.11	212.2 ± 32.82	210 ± 12.05	227.1 ± 30.72	F = 34.92 *p* = 0.001*
**Within group comparison**		P1 = 0.001*	P1 = 0.221 P2 = 0.001*	P1 = 0.157 P2 = 0.001* P3 = 0.845	P1 = 0.927 P2 = 0.001* P3 = 0.189 P4 = 0.133	
**PR(SEC)**	0.037 ± 0.008	0.054 ± 0.004	0.049 ± 0.008	0.038 ± 0.008	0.034 ± 0.007	F = 13.72 *p* = 0.001*
**Within group comparison**		P1 = 0.001*	P1 = 0.001* P2 = 0.170	P1 = 0.718 P2 = 0.001* P3 = 0.001*	P1 = 0.384 P2 = 0.001* P3 = 0.001* P4 = 0.221	
**QT (sec)**	0.073 ± 0.009	0.095 ± 0.006	0.106 ± 0.004	0.117 ± 0.025	0.088 ± 0.004	F = 16.73 *p* = 0.001*
**Within group comparison**		P1 = 0.001*	P1 = 0.001* P2 = 0.072	P1 = 0.001* P2 = 0.001* P3 = 0.06	P1 = 0.014* P2 = 0.223 P3 = 0.004* P4 = 0.001*	
**ST (mV)**	0.060 ± 0.013	0.06 ± 0.028	0.106 ± 0.045	0.101 ± 0.068	0.088 ± 0.004	F = 4.07 *p* = 0.007*
**Within group comparison**		P1 = 0.996	P1 = 0.02* P2 = 0.02*	P1 = 0.039* P2 = 0.039* P3 = 0.806	P1 = 0.413 P2 = 0.416 P3 = 0.003* P4 = 0.005*	
**R amplitude** **(mV)**	0.375 ± 0.105	0.319 ± 0.08	0.493 ± 0.056	0.405 ± 0.043	0.381 ± 0.101	F = 6.09 *p* = 0.001*
**Within group comparison**		P1 = 0.130	P1 = 0.002* P2 = 0.001*	P1 = 0.413 P2 = 0.02* P3 = 0.019*	P1 = 0.861 P2 = 0.092 P3 = 0.004* P4 = 0.519	
**T amplitude** **(mV)**	0.123 ± 0.02	0.248 ± 0.08	0.345 ± 0.09	0.325 ± 0.044	0.149 ± 0.06	F = 23.69 *p* = 0.001*
**Within group comparison**		P1 = 0.001*	P1 = 0.001* P2 = 0.002*	P1 = 0.001* P2 = 0.01* P3 = 0.477	P1 = 0.373 P2 = 0.002* P3 = 0.001* P4 = 0.001*	

**(F): **one-Way ANOVA test **, (P1)**: Difference between control group and each of studied groups, (**P2)**: difference between each group with DM, (**P3)**: difference between DM+ISO and each of studied groups, (**P4)**: difference between DM+ISO+ Metformin and all other studied groups, (*) statistically significant

**HR** is **least in DM+ISO+Metformin group followed by DM+ISO group** with inter-groups statistically significant difference {controls vs DM} and {DM versus (DM+ISO, DM+ISO+Metformin and DM+ISO+DAPA)}.

**PR interval** is **shortest in DM+ISO+DAPA group followed by controls** then **DM+ISO+Metformin group** with inter-groups statistically significant difference.

**QT interval** is **shortest in controls followed by DM+ISO+DAPA group then DM group** with inter-groups statistically significant difference.

**ST segment** is **shortest in controls and DM groups followed by DM+ISO+DAPA group** with inter-groups statistically significant difference.

**R amplitude** is highest in **DM+ISO group followed by DM+ISO+Metformin** with inter-groups statistically significant difference.

Also, **T- amplitude** is highest in **DM+ISO group followed by DM+ISO+Metformin** with inter-groups statistically significant difference.

### Results of laboratory findings between different groups

The laboratory findings between the 5 groups were listed in Tables 2, 3.

**Table 2:** Laboratory findings of studied groups

	Normal N = 10	DM N = 10	DM+ISO N = 10	DM+ISO+ Metformin N = 10	DM+ISO+DAPA N = 10	Test of significance
**IL-10(Pg/ml)**	69.89 ± 110.29	84.52 ± 15.44	154.49 ± 19.39	394.29 ± 36.92	657.63 ± 49.09	F = 405.84 *p* = 0.001*
**Within group comparison**		P1 < 0.001*	P1 < 0.001* P2 = 0.009*	P1 < 0.001* P2 < 0.001* P3 < 0.001*	P1 < 0.001* P2 < 0.001* P3 < 0.001* P4 < 0.001*	
**IL-6(Pg/ml)**	89.69 ± 21.43	785.31 ± 34.31	623.06 ± 55.72	444.17 ± 49.75	197.63 ± 33.48	F = 500.03 *p* = 0.001*
**Within group comparison**		P1 < 0.001*	P1 < 0.001* P2 = 0.001*	P1 < 0.001* P2 < 0.001* P3 < 0.001*	P1 < 0.001* P2 < 0.001* P3 < 0.001* P4 < 0.001*	
**TNF(Pg/ml)**	81.27 ± 23.18	770.01 ± 48.30	563.58 ± 45.58	406.05 ± 27.27	206.82 ± 22.52	F = 610.12 *p* = 0.001*
**Within group comparison**		P1 < 0.001*	P1 < 0.001* P2 = 0.001*	P1 < 0.001* P2 < 0.001* P3 < 0.001*	P1 < 0.001* P2 < 0.001* P3 < 0.001* P4 < 0.001*	
**CTNI(Pg/ml)**	64.90 ± 20.38	934.04 ± 54.09	760.33 ± 36.85	494.27 ± 62.36	185.49 ± 40.34	F = 665.24 *p* = 0.001*
**Within group comparison**		P1 < 0.001*	P1 < 0.001* P2 = 0.001*	P1 < 0.001* P2 < 0.001* P3 < 0.001*	P1 < 0.001* P2 < 0.001* P3 < 0.001* P4 < 0.001*	
**Troponin C(mg/dl)**		103.25 ± 13.39	266.23 ± 13.88	290.85 ± 35.82	223.09 ± 18.54	153.71 ± 11.91
**Within group comparison**		P1 < 0.001*	P1 < 0.001* P2 = 0.01*	P1 < 0.001* P2 < 0.001* P3 < 0.001*	P1 < 0.001* P2 < 0.001* P3 < 0.001* P4 < 0.001*	

**(P1)**: Difference between control group and each of studied groups, **(P2:) **difference between each group with DM, **(P3:)** difference between DM+ISO and each of studied groups, **(P4)** difference between DM+ISO+Metformin and all other studied groups, **(P5:) **difference between each group and DM+ISO+DAPA, (*) statistically significant

**Table 3:** Laboratory findings of studied groups (continued)

	**Normal** **N = 10**		**DM** **N = 10**	**DM+ISO** **N = 10**	**DM+ISO+** **Metformin** **N = 10**	**DM+ISO+DAPA** **N = 10**	**Test of significance**
**Glucose(mg/dl)**	105.73 ± 20.88		296.45 ± 32.77	246.22 ± 19.48	179.21 ± 16.36	136.94 ± 7.27	F = 138.48 *p* = 0.001*
**Within group comparison**			P1 < 0.001*	P1 < 0.001* P2 = 0.009*	P1 < 0.001* P2 < 0.001* P3 < 0.001*	P1 = 0.002* P2 < 0.001* P3 < 0.001* P4 < 0.001*	
**T.G(mg/dl)**	84.78 ± 12.68		156.88 ± 11.08	183.39 ± 5.31	155.47 ± 10.29	123.92 ± 10.84	F = 133.30 *p* = 0.001*
**Within group comparison**			P1 < 0.001*	P1 < 0.001* P2 = 0.001*	P1 < 0.001* P2 = 0.763 P3 < 0.001*	P1 < 0.001* P2 < 0.001* P3 < 0.001* P4 < 0.001*	
**HDL(mg/dl)**	45.57 ± 2.02		51.09 ± 1.75	47.90 ± 1.02	47.95 ± 3.06	48.17 ± 3.19	F = 6.92 *p* = 0.001*
**Within group comparison**			P1 < 0.001*	P1 = 0.032* P2 = 0.004*	P1 = 0.029* P2 = 0.005* P3 = 0.962	P1 = 0.018* P2 = 0.008* P3 = 0.800 P4 = 0.836	
LDL**(mg/dl)**		40.72 ± 8.85	178.29 ± 12.33	206.26 ± 35.71	139.18 ± 16.40	75.22 ± 10.97	F = 126.55 *p* = 0.001*
**Within group comparison**			P1 < 0.001*	P1 < 0.001* P2 = 0.001*	P1 < 0.001* P2 < 0.001* P3 < 0.001*	P1 < 0.001* P2 < 0.001* P3 < 0.001* P4 < 0.001*	
VLDL**(mg/dl)**		16.96 ± 2.54	30.66 ± 1.70	36.68 ± 1.06	31.26 ± 1.84	24.97 ± 2.77	F = 126.55 *p* = 0.001*
**Within group comparison**			P1 < 0.001*	P1 < 0.001* P2 = 0.001*	P1 = 0.525 P2 < 0.001* P3 < 0.001*	P1 < 0.001* P2 < 0.001* P3 < 0.001* P4 < 0.001*	

**(P1):** Difference between control group and each of studied groups, **(P2:) **difference between each group with DM, **(P3:)** difference between DM+ISO and each of studied groups, **(P4)** difference between DM+ISO+Metformin and all other studied groups, **(P5:) **difference between each group and DM+ISO+DAPA, (*) statistically significant

All markers (**IL-10, IL-6, TNF, CTNI and Troponin C)** were higher in **DM+ISO group** than other groups of study (394.29±36.92, 444.17 ± 49.75, 406.05 ± 27.27, 494.27 ± 62.36, 290.85 ± 35.82 respectively) which revealed the successful induced MI without cardiac protection.

On the other hand, IL-10, IL-6, TNF, CTNI and **Troponin** C were lowest in (**control)** group (69.89±110.29, 89.69 ± 21.43, 81.27 ± 23.18, 64.90 ± 20.38, 103.25 ± 13.39) followed by **(DM+ISO+DAPA)** group (657.63±49.09,197.63±33.48,206.82±22.52,185.49±4Metformin0.9±18.54) then **(DM+ISO+Metformin)** group (290.85±35.82,444.17±49.75,406.05±27.27,494.27±62.36,394.29±36.92) with inter-groups statistically significant difference in-between DAPA and Metformin groups (*p* < 0.001*) which represent the successful cardio protective effect of DAPA over Metformin.

**glucose** is lowest in controls (105.73±20.88) followed by **DM+ISO+DAPA** (136.94±7.27) group then **DM+ISO+Metformin **(179.21±16.36) group with inter-groups statistically significant difference in between all included groups which ensure the successful induction of DM among study groups and better control of blood glucose level by DAPA than **Metformin**.

**Triglycerides (T.G), LDL and VLDL** were lowest in **controls** followed by **DM+ISO+DAPA** group then **DM+ISO+Metformin, While HDL was** lowest in **controls** followed by **DM+ISO** group then **DM+ISO+ Metformin** group with inter-groups statistically significant, so the incidence of metabolic syndrome was lowest among DAPA group.

### Histopathological results

**Fig. 4:**
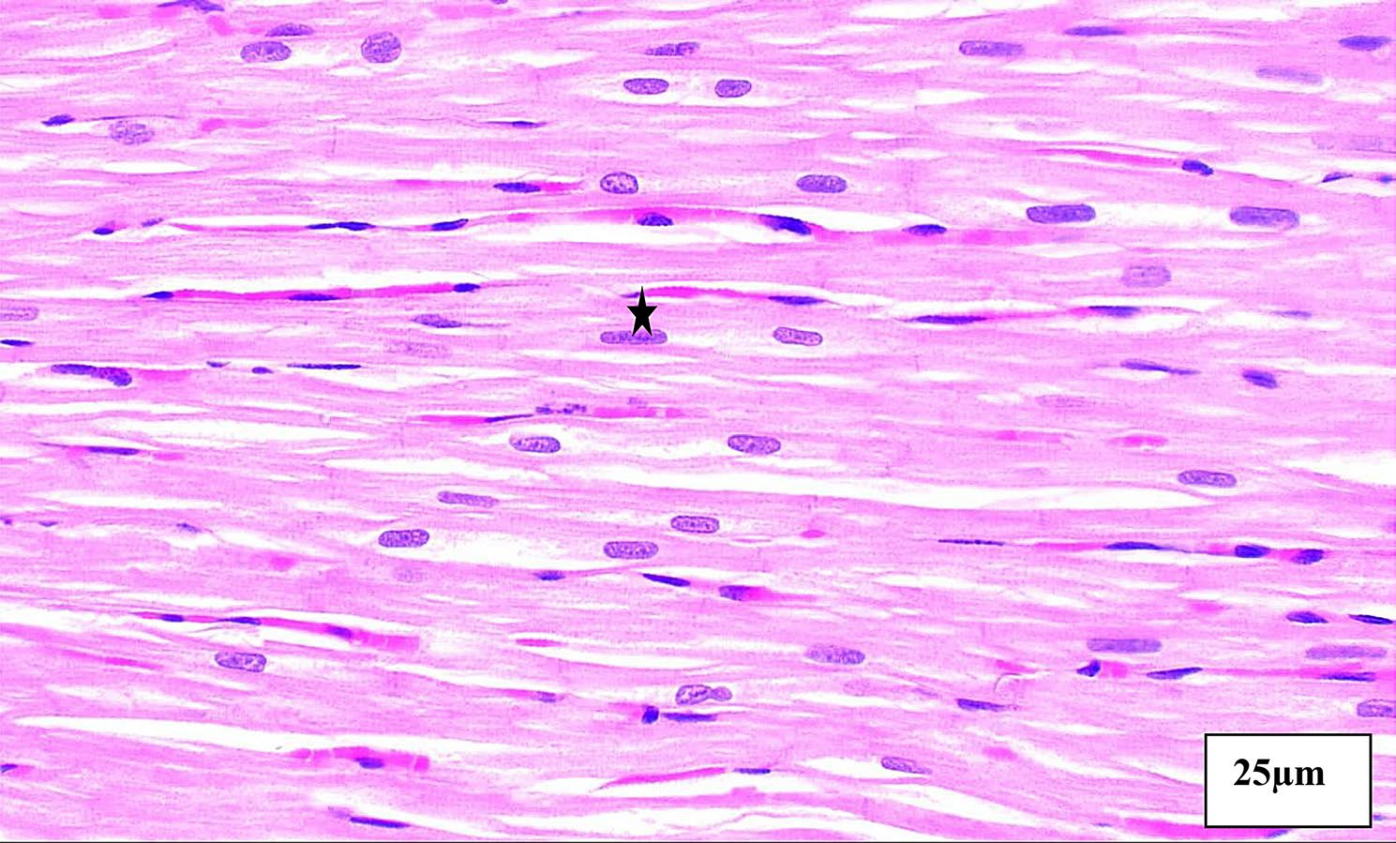
Aphotomicrogragh of a paraffin section in the ventricle of **control group** showing the cardiac muscle fibers are branching and anastomosing with minimal interstitial spaces containing fibroblast cells with flat nuclei (**stars**). The cardiomyocytes have acidophilic faintly striated cytoplasm (**thin arrows**) and oval central vesicular nuclei (**thick arrows**). (**H&E x400**)

**Fig. 5:**
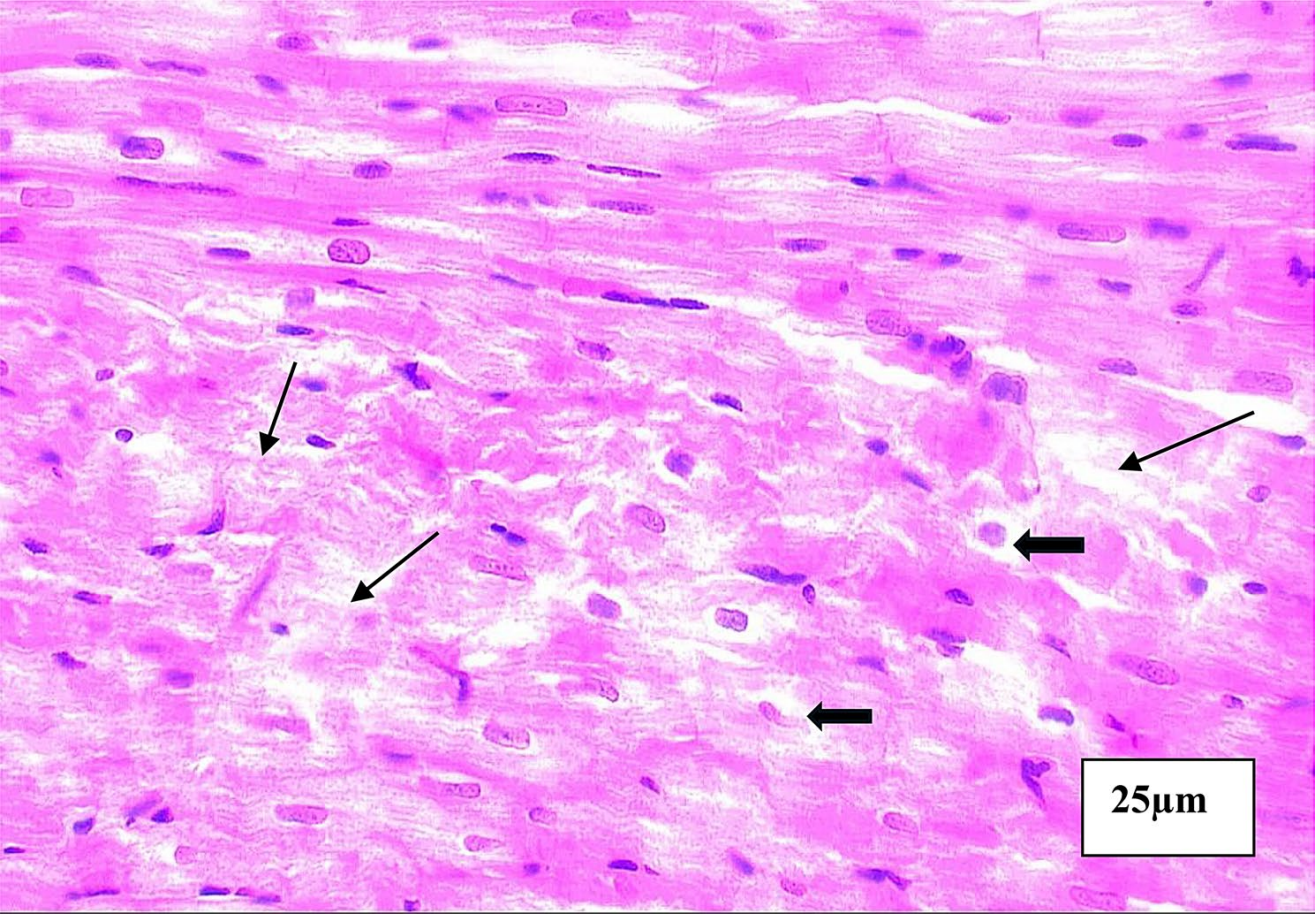
Aphotomicrogragh of a paraffin section in the ventricle of **DM group** showing branched disorganized cardiac muscle fibers (**thin arrows**), some cardiomyocytes with vacuolated cytoplasm (**thick arrows**). (HX and E X 400)

**Fig. 6:**
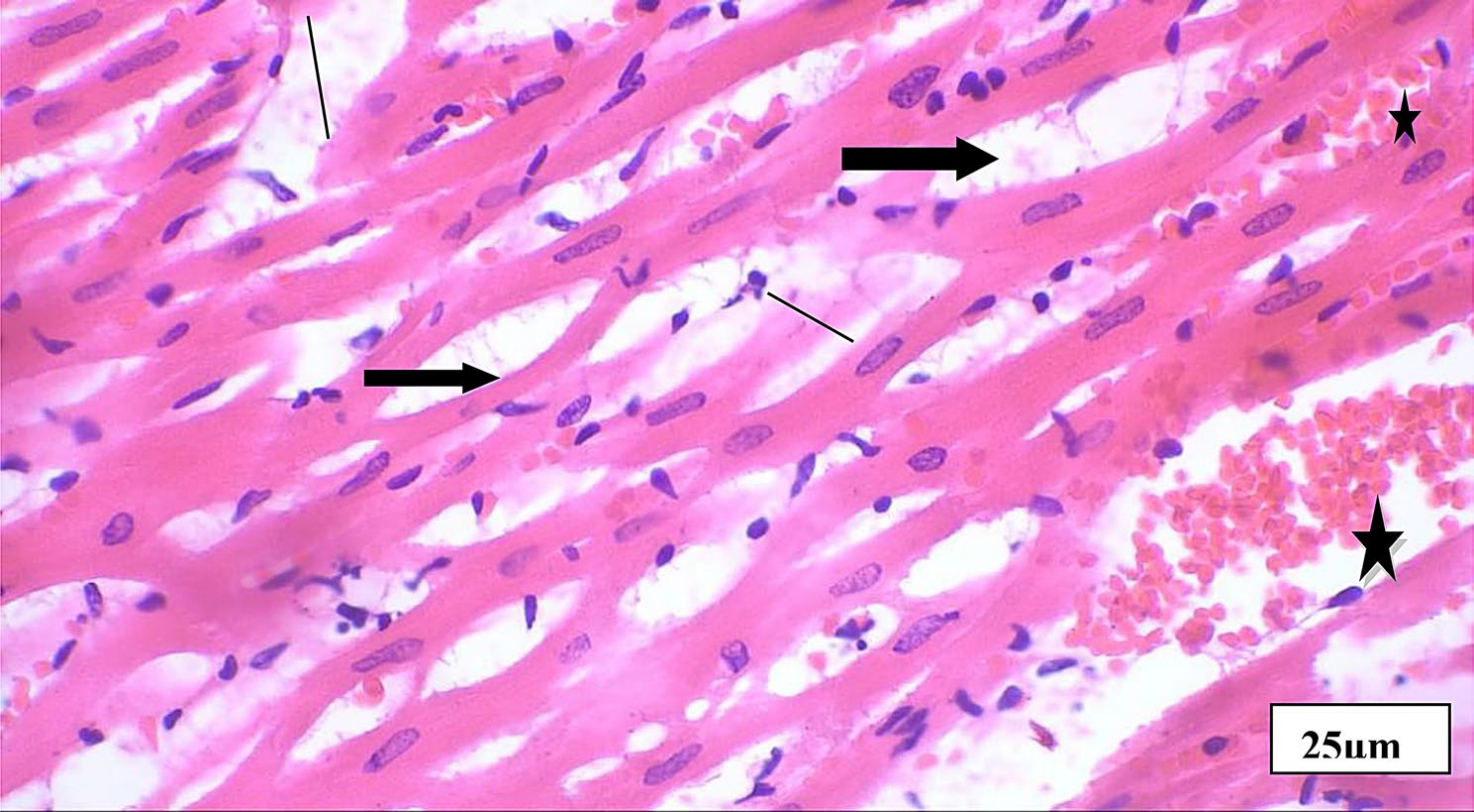
Aphotomicrogragh of a paraffin section in the ventricle of **MI group** showing massive disruption of the cardiac muscl fibers (**thin arrow**) and massive interstitial hemorrhage (stars) and edema fluid between fibers (**thick arrows**) (**HX and E X 400**)

**Fig. 7:**
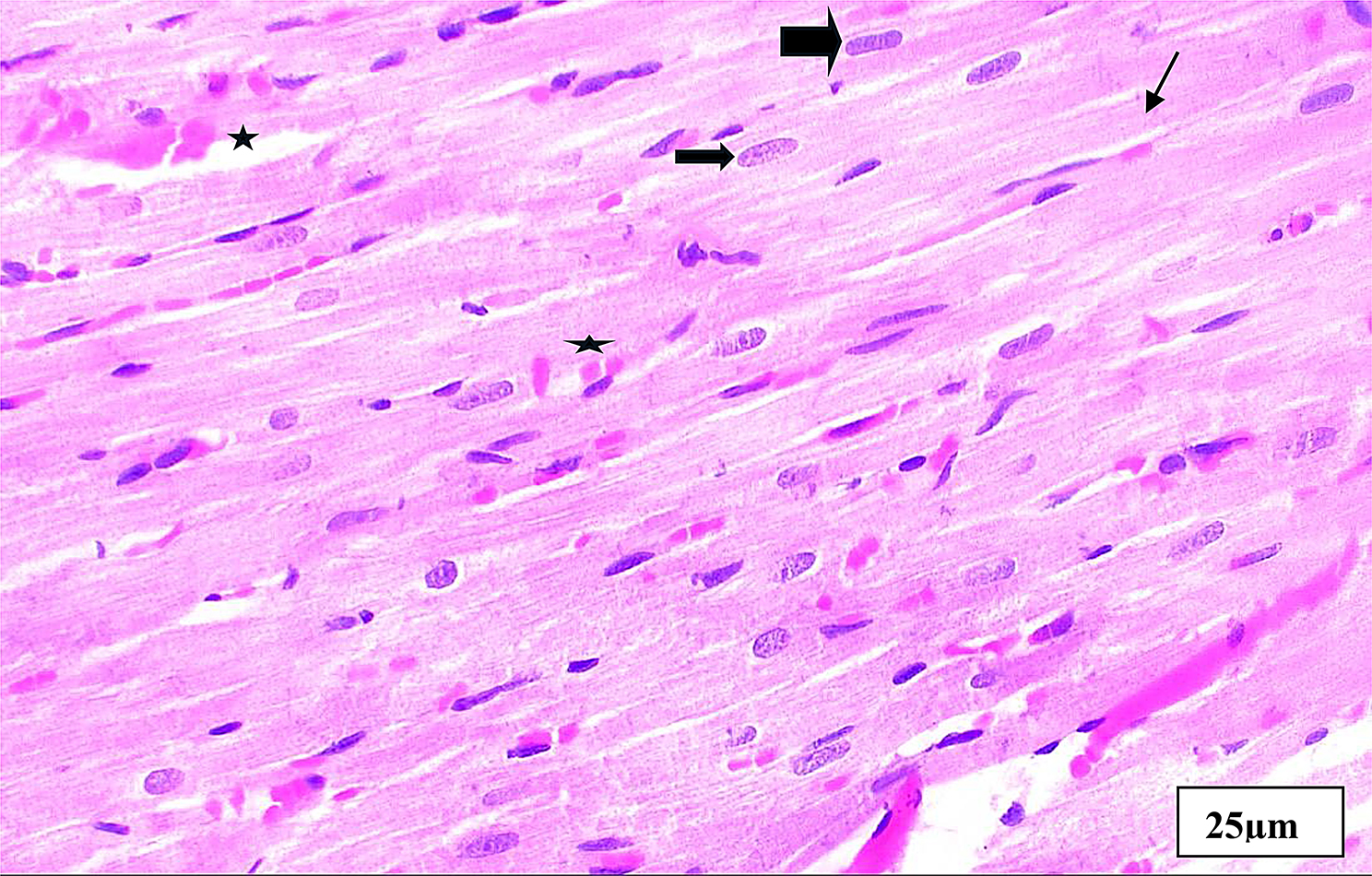
Aphotomicrogragh of a paraffin section in the ventricle of **DM + metformin** group illustrating improved cardiomyocytes histology with acidophilic cytoplasm, central nuclei (**thick arrows**) and intracellular cross striations (**thin arrows**). Note minimal interstiaial hemorrage (**stars**) compared to DM group. (**H&E x400**)

**Fig. 8:**
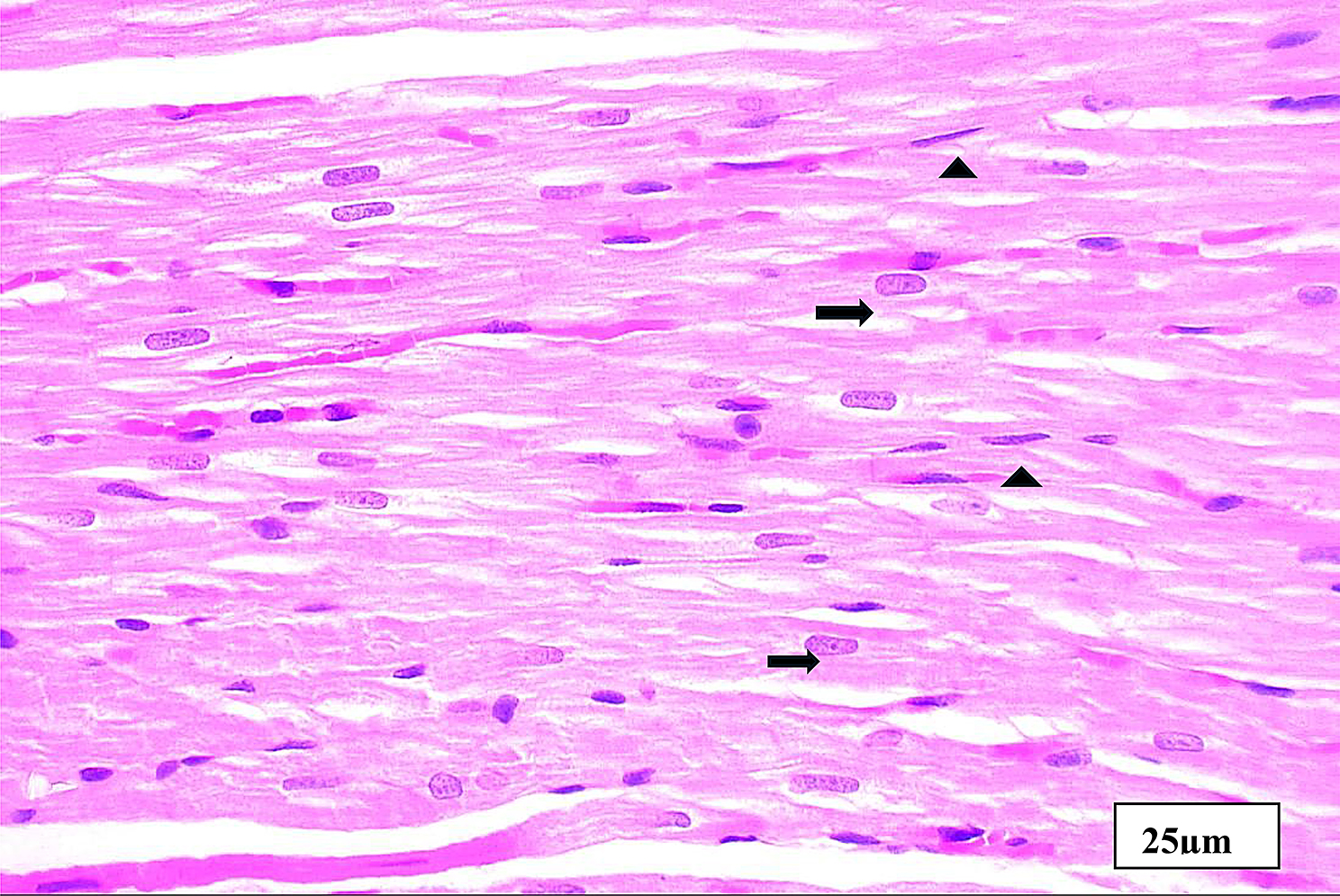
Aphotomicrogragh of a paraffin section in the ventricle of **DM+DAPA** group demonstrating improved cardiac muscl fibers restoring nearly the normal structure with acidophilic cytoplasm, central nuclei (**thick arrows**), and minimal interstitial spaces containing fibroblast cells with flat nuclei (**arrow head**). (**H&E x400**)

The Histopathological findings between the 5 groups were listed in Table 4 and Figs. 4, 5, 6, 7, 8.

**Table 4:** Area degeneration and oedema among studied groups

	**Normal** **N = 10**		**DM** **N = 10**	**DM+ISO** **N = 10**	**DM+ISO+** **Metformin** **N = 10**	**DM+ISO+DAPA** **N = 10**	**Test of significance**
**Area Degeneration (%)**		0.221 ± 0.07	3.49 ± 0.35	6.45 ± 0.66	0.97 ± 0.006	0.813 ± 0.005	F = 588.01 *p* = 0.001*
**Within group comparison**			P1 < 0.001*	P1 < 0.001* P2 = 0.001*	P1 < 0.001* P2 < 0.001* P3 < 0.001*	P1 < 0.001* P2 < 0.001* P3 < 0.001* P4 = 0.303	
**Area Edema(%)**		0.873 ± 0.042	13.84 ± 1.19	23.39 ± 2.21	6.07 ± 0.06	4.82 ± 0.10	F = 630.63 *p* = 0.001*
**Within group comparison**			P1 < 0.001*	P1 < 0.001* P2 = 0.001*	P1 < 0.001* P2 < 0.001* P3 < 0.001*	P1 < 0.001* P2 < 0.001* P3 < 0.001* P4 = 0.017*	

(P1): Difference between control group and each of studied groups, (P2:) difference between each group with DM, (P3:) difference between DM+ISO and each of studied groups, (P4) difference between DM+ISO+Metformin and all other studied groups, (P5:) difference between each group and DM+ISO+DAPA, (*) statistically significant

Percentage of **area of edema and degeneration** is lowest in controls followed by DM+ISO+DAPA group then DM+ISO+Metformin group when compared with DM and DM+ISO groups which revealed the cardio protective effect of DAPA and Metformin

On the other hand, **DAPA** group showed lower areas of degeneration and areas of edema than **Metformin** group

## Discussion

The main cause of morbidity and mortality among people with Type 2 Diabetes mellitus (T2DM) is heart disease. Diabetic cardiomyopathy is a rare form of cardiomyopathy that causes heart failure in approximately 20% of individuals with type 2 diabetes and accounts for more than half of diabetic patient deaths [9].

According to recent research, anti-diabetic medications have an impact on diabetic patients’ cardiovascular risk that is mostly unrelated to their benefits on decreasing blood sugar [9].

Metformin, which is frequently used for type-2 diabetes, stimulates hepatic and muscle adenosine monophosphate -activated protein kinase (AMPK), which in turn makes insulin more sensitive.

Another class of antidiabetic medications, known as sodium glucose transporter-2 (SGLT2) inhibitors, includes dapagliflozin. Since cardiac tissues lack SGLT-2, dapagliflozin’s anti-inflammatory effects happen without SGLT-2‘s assistance. Increasing the (M2/M1) phenotypic macrophage ratio is the primary mechanism by which dapagliflozin reduces inflammation and induces an increase in anti-inflammatory cytokine mRNA levels like IL-10 and a decrease in inflammatory cytokine mRNA levels like IL-1β and IL-6 [10].

This is comparative study, which was housed at the animal house, Mansoura Experimental Research Center (MERC). Fifty adult male albino rats were included in this study and there were further randomly divided into Group- I: (Normal), Group –IIa: (DM), Group-IIb: (DM+ISO), Group –IIc: (DM+ISO+Metformin), Group- IId: (DM+ISO+DAPA).

Our results denoted that metformin treatment could have desirable effect on HR, PR interval, **R amplitude and T amplitude. While, Dapagliflozin** treatment could have more desirable effect on PR interval, QT interval, **ST segment, R amplitude and T- amplitude.**

In harmony with our findings, *Ibrahim et al., 2022* found significant improvements in ECG, such as a slower heart rate, longer R-R, a shorter QT interval, a larger R wave amplitude, and a lower ST segment elevation after Metformin treatment when compared to ISO group [5]. This reduced the amount of oxygen consumed by the heart and the degree of necrosis. The anti-inflammatory, anti-apoptotic, and antioxidant properties of metformin were cited as the explanation for these cardio-protective benefits [11].

Additionally, in the Ibrahim et al. (2022) trial, all recorded ECG parameters showed significant improvements in the dapagliflozin-treated group when compared to the ISO group. Furthermore, a significantly larger **R wave amplitude** was observed in the group treated with Dapagliflozin than in the group treated with Metformin. This may be as a result of dapagliflozin’s ability to lessen cardiomyocyte apoptosis, lower mitochondrial ROS generation, and lessen cardiac mitochondrial dysfunction. Additionally, compared to the group treated with metformin, the HR of the dapagliflozin group was considerably lower. Lastly, the **QT interval** was significantly reduced in the group that received dapagliflozin treatment [5].

Our results are supported by Durak et al. (2018), who found that dapagliflozin therapy decreased the amplification of depressed voltage-gated K±channel currents, improving delayed cardiac repolarization and the prolonged QT-interval in the ECG. [12]. Furthermore, a study by Juttla et al. (2024) demonstrated that the QT interval was considerably shorter with dapagliflozin prophylaxis, and that QT interval prolongation is linked to steadily declining glucose tolerance in type 2 diabetes [13].

Contrary to what we found, a randomized study using human volunteers in good health revealed that the QT interval was unaffected using SGLT2 inhibitors [14]. The results of a trial that employed supra-therapeutic doses of dapagliflozin, however, showed no clinically significant impact on the QT interval [15].

Also, the present study showed that **IL-10, IL-6, TNF, CTNI and Troponin C** were higher in **DM+ISO group** than other groups of study which revealed the successful induced MI without cardiac protection.

On the other hand, IL-10, IL-6, TNF, CTNI and Troponin C were lowest in **control group** followed by **(DM+ISO+DAPA)** group then **(DM+ISO+Metformin)** group with inter-groups statistically significant difference in-between DAPA and Metformin groups (p < 0.001*) which represent the successful cardio protective effect of DAPA over Metformin.

Similarly, Ibrahim et al. (2022) demonstrated that, in comparison to the ISO-induced MI control group, therapy with metformin, sitagliptin, and dapagliflozin was linked to a significant decrease in cTn-1, with mean values of 783.6 ± 13.25, 767.0 ± 20.01, and 750.1 ± 14.26, respectively **(p < 0.001)**. The cTn-I level was significantly lower in the dapagliflozin-treated group than in the metformin-treated group **(p < 0.001)** [5].

**Lee et al. (2017)** showed that dapagliflozin’s capacity to promote macrophage polarization toward an anti-inflammatory phenotype resulted in elevated IL-10 and an enhanced ratio of M2/M1 phenotypic macrophages [16]. **Al Rasheed et al. (2018)** reported that metformin’s capacity to reduce cTn-I in ISO-induced MI via blocking NF-κB activation in vascular smooth muscle cells and endothelial cells [17].

After four weeks of dapagliflozin exposure, **Hussein et al. (2020)** found that cardiac caspase-3, sympathetic activity, and myocardial tyrosine hydroxylase activity were all reduced, along with inflammatory TNF-α, TGF-β, and TGF-β cytokines [18].

In our study, **glucose** is lowest in controls followed by **DM+ISO+DAPA** group then **DM+ISO+Metformin** group with inter-groups statistically significant difference in between all included groups which ensure the successful induction of DM among study groups and better control of blood glucose level by DAPA than **Metformin**.

**Triglycerides (T.G), LDL and VLDL** were lowest in **controls** followed by **DM+ISO+DAPA** group then **DM+ISO+Metformin, While HDL was** lowest in **controls** followed by **DM+ISO** group then **DM+ISO+ Metformin** group with inter-groups statistically significant, so the incidence of metabolic syndrome was lowest among DAPA group.

According to a study by **El-Shafey et al. (2022)**, as compared to the diabetic group, therapy with DAPA for eight weeks led to a 1.55-fold **(p < 0.0001)** increase in serum insulin levels and a 42.5% significant drop **(p < 0.0001)** in blood glucose levels [19]^.^ Additionally, **Juttla et al. (2024)** demonstrated that dapagliflozin had notable euglycemic effects. This is consistent with its well-established glycemic actions that are independent of insulin [13].

In keeping with our results, **Osorio and his colleagues** report that in a mouse model of steatohepatitis, metformin improves lipid metabolism by preventing and correcting inflammation and steatosis [20].

Another review reinforced the association between AMPK and metformin by confirming that AMPK’s inhibitory phosphorylation of acetyl-CoA carboxylase is essential for controlling lipid metabolism [21].

Interestingly, the present study showed Percentage of **area of edema and degeneration** is lowest in controls followed by **DM+ISO+DAPA** group then **DM+ISO+Metformin** group when compared with **DM** and **DM+ISO** groups which revealed the cardio protective effect of DAPA and Metformin, on the other hand **DAPA** group showed lower areas of degeneration and areas of edema than **Metformin** group.

In harmony, **El-Shafey et al. (2022)** showed that cardiac myocytes in slices from diabetic rat hearts were asymmetrical, with mild chronic inflammatory cells in the sub pericardium, myocardial fiber disarray, and myocardial fiber necrosis. Apoptotic myocytes with pyknotic nuclei, hyper eosinophilic cytoplasm, and an increase in intermyocytes, interstitial edema, and perivascular chronic inflammatory cells were also discovered. The group receiving DM+ DAPA treatment appears to have substantial damage, while the group receiving DM+ liraglutide treatment appears to have mild damage [19].

The efficacy of metformin and dapagliflozin to reduce doxorubicin-induced acute cardiotoxicity in Wistar rats was evaluated in a prior investigation. Intermuscular edema (*p* < 0.001) and cardiomyocyte production (*p* < 0.001) were significantly reduced in the metformin and dapagliflozin-treated groups compared to the non-treated group [22].

Our research demonstrated that **dapagliflozin** can be used as a first-line oral antidiabetic medication in place of **metformin**. Further research should include other medications in the same class to guarantee the same results, as it is still unclear whether the effects seen are specific to dapagliflozin alone or if they also apply to other medications in the same class.

## Conclusion

The results of this trial demonstrate that dapagliflozin, an SGLT2 inhibitor, has an important cardioprotective role in type 2 diabetes, including antifibrotic and antiarrhythmic effects. Metformin did not have the latter effect.

They may also take the place of metformin as the first-choice oral antidiabetic medication. It’s still unclear if these effects are specific to dapagliflozin or if they also apply to other medications in the same family.

Future research should aim to close this knowledge gap and make it easier to apply these findings in real-world clinical settings [23].

## Data Availability

The data from this study can be obtained upon request from the corresponding author.

## Abbreviation

AMPK: Adenosine monophosphate activated protein kinase
DAPA: Dapagliflozin
E: Eosin
ECG: Electrocardiogram
GLP-1: Glucagon-like peptide-1
HX: Hematoxylin
IL: Interleukin
ISO: Isoprenaline
MERC: Mansoura Experimental Research Center
MI: Myocardial infarction
NFκB: Nuclear factor kappa-light-chain-enhancer of activated B
NO: Nitric oxide
SGLT2: Sodium-glucose co-transporter
STZ: Streptozotocin
T2DM: Type 2 diabetes mellitus
TNF: Tumor necrosis factor

## References

[1] Cole JB, Florez JC. Genetics of diabetes mellitus and diabetes complications. Nat Rev Nephrol. 2020, Jul;16(7):377–90. 32398868 10.1038/s41581-020-0278-5 PMC9639302

[2] Morton JI, Lazzarini PA, Shaw JE, Magliano DJ. Trends in the incidence of hospitalization for major diabetes-related complications in people with type 1 and type 2 diabetes in Australia, 2010-2019. Diabetes Care. 2022, Apr, 1;45(4):789–97. 35085387 10.2337/dc21-2268

[3] Ma CX, Ma XN, Guan CH, Li YD, Mauricio D, Fu SB. Cardiovascular disease in type 2 diabetes mellitus: progress toward personalized management. Cardiovasc Diabetol. 2022, May, 14;21(1):74. 35568946 10.1186/s12933-022-01516-6 PMC9107726

[4] Ezeani IU, Eregie A, Ohwovoriole AE. Anti-diabetic agents and the potentials for reducing cardiovascular risks in type-2 diabetes mellitus. Ann Health Res (The J The Med Dent Consultants Assoc Nigeria) ,OOUTH, Sagamu, Nigeria. 2021, Sep, 27;7(3):208–26.

[5] Ibrahim MM, Khedr MM, Morsy MH, Badae NM, Elatrebi S. A comparative study of the cardioprotective effect of metformin, sitagliptin and dapagliflozin on isoprenaline induced myocardial infarction in non-diabetic rats. Bull The Natl Res Cent. 2022;46(1):123.

[6] Castoldi G, Carletti R, Ippolito S, Colzani M, Pelucchi S, Zerbini G, et al. Cardioprotective effects of sodium glucose cotransporter 2 inhibition in angiotensin II-dependent hypertension are mediated by the local reduction of sympathetic activity and inflammation. Int J Mol Sci. 2023, Jun, 27;24(13):10710. 37445888 10.3390/ijms241310710 PMC10341774

[7] Szkudelski T. Streptozotocin-nicotinamide-induced diabetes in the rat. Characteristics of the experimental model. Exp Biol Med. 2012, May;237(5):481–90. 10.1258/ebm.2012.011372 22619373

[8] Suvarna S, Layton C, Bancroft J. The hematoxylins and eosin. Bancroft’s theory and practice of histological techniques. 7th. London, UK: Churchill Livingstone; 2013. p. 172–86.

[9] Patti AM, Rizvi AA, Giglio RV, Stoian AP, Ligi D, Mannello F. Impact of glucose-lowering medications on cardiovascular and metabolic risk in type 2 diabetes. J Clin Med. 2020, Mar, 26;9(4):912. 32225082 10.3390/jcm9040912 PMC7230245

[10] Belančić A, Klobučar S. Sodium-glucose Co-transporter 2 inhibitors as a powerful cardioprotective and renoprotective tool: overview of clinical trials and mechanisms. Diabetology. 2023, Jul, 4;4(3):251–58.

[11] Al-Kuraishy H, Hussein R. Caspase-3 levels (CASP-3) in doxorubicin induced-cardiotoxicity: role of metformin pretreatment. Res J Oncol. 2017;1(1):4.

[12] Durak A, Olgar Y, Degirmenci S, Akkus E, Tuncay E, Turan B. A SGLT2 inhibitor dapagliflozin suppresses prolonged ventricular-repolarization through augmentation of mitochondrial function in insulin-resistant metabolic syndrome rats. Cardiovasc Diabetol. 2018, Nov, 17;17(1):144. 30447687 10.1186/s12933-018-0790-0 PMC6240275

[13] Juttla PK, Chege BM, Mwangi PW, Bukachi F. Dapagliflozin pretreatment prevents cardiac electrophysiological changes in a diet and streptozotocin induction of type 2 diabetes in rats: a potential new first-line? J Exp Pharmacol. 2024, Dec;31:123–33. 10.2147/JEP.S443169 PMC10961018 38525051

[14] Ring A, Brand T, Macha S, Breithaupt-Groegler K, Simons G, Walter B, et al. The sodium glucose cotransporter 2 inhibitor empagliflozin does not prolong QT interval in a thorough QT (TQT) study. Cardiovasc Diabetol. 2013, Apr, 24;12(1):70. 23617452 10.1186/1475-2840-12-70 PMC3648489

[15] Carlson GF, Tou CK, Parikh S, Birmingham BK, Butler K. Evaluation of the effect of dapagliflozin on cardiac repolarization: a thorough QT/QTc study. Diabetes Ther. 2011, Sep;2(3):123–32. 22127822 10.1007/s13300-011-0003-2 PMC3173598

[16] Lee TM, Chang NC, Lin SZ. Dapagliflozin, a selective SGLT2 inhibitor, attenuated cardiac fibrosis by regulating the macrophage polarization via STAT3 signaling in infarcted rat hearts. Free Radical Biol & Med. 2017, Mar;1(104):298–310. 10.1016/j.freeradbiomed.2017.01.035 28132924

[17] Al-Rasheed NM, Al-Rasheed NM, AL-Rabeeah DA, AL-Barrak HS, AL-Salman SA, Ibrahim SA, et al. Possible protective mechanisms exerted by metformin or metformin and vitamin E in isoproterenol-induced cardiac injury. J Cellular Biochem. 2018, May;119(5):3903–12. 29165830 10.1002/jcb.26530

[18] Hussein AM, Eid EA, Taha M, Elshazli RM, Bedir RF, Lashin LS. Comparative study of the effects of GLP1 analog and SGLT2 inhibitor against diabetic cardiomyopathy in type 2 diabetic rats: possible underlying mechanisms. Biomedicines. 2020, Feb, 25;8(3):43. 32106580 10.3390/biomedicines8030043 PMC7175346

[19] El-Shafey M, El-Agawy MS, Eldosoky M, Ebrahim HA, Elsherbini DM, El-Sherbiny M, et al. Role of dapagliflozin and liraglutide on diabetes-induced cardiomyopathy in rats: implication of oxidative stress, inflammation, and apoptosis. Front. Endocrinol. 2022, Mar, 18;13:862394. 10.3389/fendo.2022.862394 PMC8972060 35370937

[20] Osorio-Llanes E, Villamizar-Villamizar W, Ospino Guerra MC, Díaz-Ariza LA, Castiblanco-Arroyave SC, Medrano L, et al. Effects of metformin on ischemia/reperfusion injury: new evidence and mechanisms. Pharmaceuticals. 2023, Aug, 9;16(8):1121. 37631036 10.3390/ph16081121 PMC10459572

[21] Lee M, Katerelos M, Gleich K, Galic S, Kemp BE, Mount PF, et al. Phosphorylation of acetyl-CoA carboxylase by AMPK reduces renal fibrosis and is essential for the anti-fibrotic effect of metformin. J Am Soc Nephrol. 2018, Sep, 1;29(9):2326–36. 29976587 10.1681/ASN.2018010050 PMC6115654

[22] Satyam SM, Bairy LK, Shetty P, Sainath P, Bharati S, Ahmed AZ, et al. Metformin and dapagliflozin attenuate doxorubicin-induced acute cardiotoxicity in Wistar rats: an electrocardiographic, biochemical, and histopathological approach. Cardiovasc Toxicol. 2023, Feb;23(2):107–19. 36790727 10.1007/s12012-023-09784-8 PMC9950216

[23] Foretz M, Guigas B, Viollet B. Metformin: update on mechanisms of action and repurposing potential. Nat Rev Endocrinol Endocrinol. 2023, Aug;19(8):460–76. 10.1038/s41574-023-00833-4 PMC10153049 37130947

